# Supplementary figures and images for: The mechanism and effectiveness of mindfulness-based intervention for reducing the psychological distress of parents of children with autism spectrum disorder: A protocol of randomized control trial of ecological momentary intervention and assessment
Source: PLoS One. 2023 Sep 13;18(9):e0291168. doi: 10.1371/journal.pone.0291168 (PMC10499232; doi:10.1371/journal.pone.0291168)

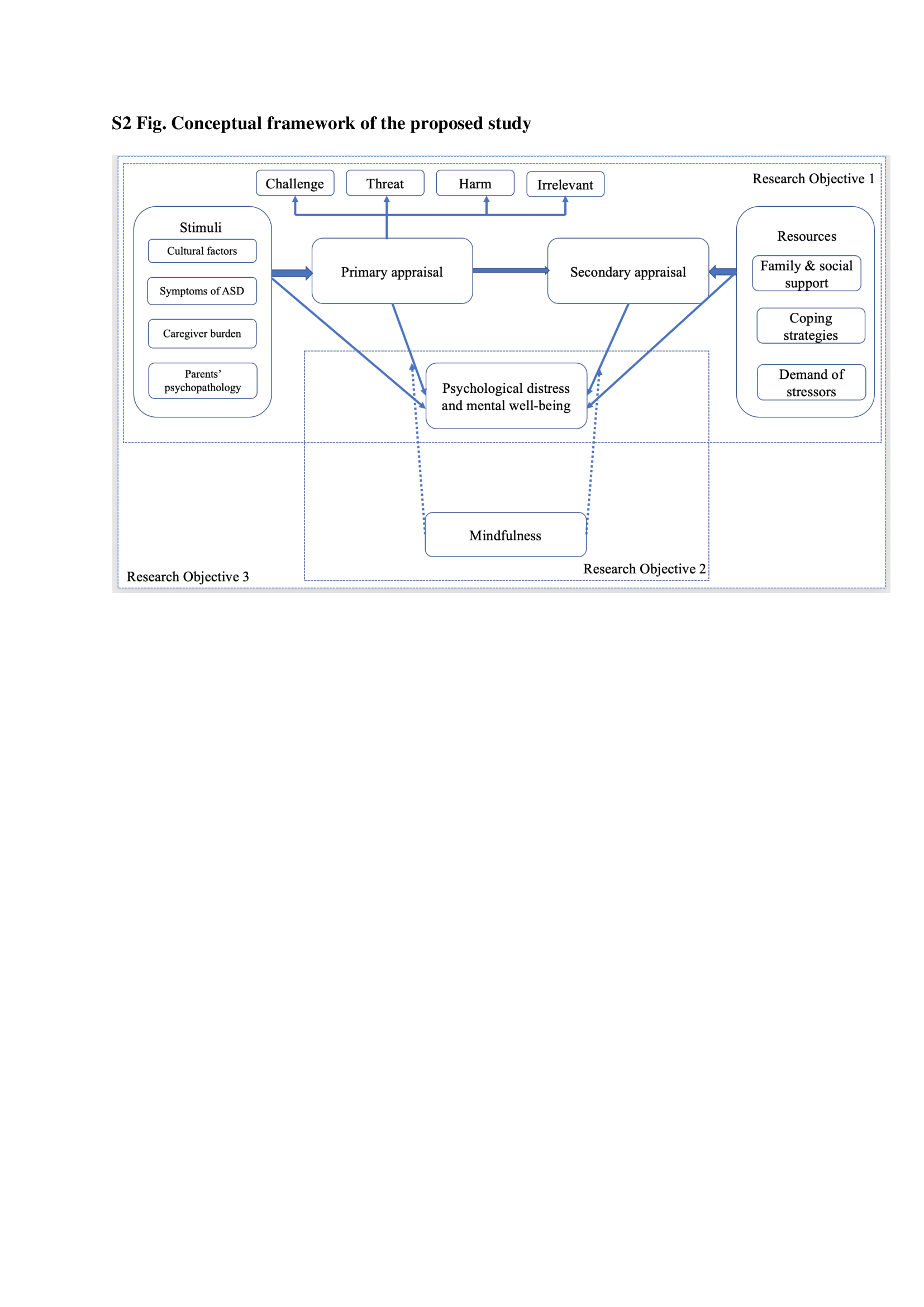

Supplement: S1 Fig — (TIF) [file pone.0291168.s001.tif]
