# Supplementary material for: The mechanism and effectiveness of mindfulness-based intervention for reducing the psychological distress of parents of children with autism spectrum disorder: A protocol of randomized control trial of ecological momentary intervention and assessment
Source: PLoS One. 2023 Sep 13;18(9):e0291168. doi: 10.1371/journal.pone.0291168 (PMC10499232; doi:10.1371/journal.pone.0291168)
Supplement: S2 File — (PDF) [file pone.0291168.s003.pdf]

LINGNAN UNIVERSITY

Application by Academic Staff for Research Ethics and Safety Approval
for project involving human participants

Applicant (PI) is required to:
1) Send the completed form to reach the Office of Research and Knowledge Transfer at least 2 weeks before conducting data collection.
2) Attach the research proposal to this application.
3) Attach the interview questions or protocol in English and original language (if any).
4) Attach the questionnaire in English and original language (if any).
5) Attach an informed consent form with a statement tailored to the study for any research that involves human participants.

For student research, please refer to the School of Graduate Studies website for the procedures and application form: https://www.ln.edu.hk/sgs/research-postgraduate-studies/Students/guidebook-for-rpg-students (Appendix E).

Please ☑ the appropriate boxes.

Part A – To be completed by Principal Investigator

1. Principal Investigator (PI)

**Staff Name:**	WANG Qi
**Post:**	Postdoctoral fellow/Research Assistant Professor
**Department:**	School of Graduate Studies
**Title of Research Project:**	The mechanism and effectiveness of mindfulness-based intervention for reducing the psychological distress of parents of children with autism spectrum disorder: An ecological momentary intervention and randomized controlled trial
**Funding obtained/applied for the project?**	☑ Yes ☐ No
**If yes, what funding scheme:**	General Research Fund
**Is the funding:**	☐ obtained ☑ being applied for

2. Details of procedures to be used in the research

1. Background

Autism spectrum disorder (ASD) is defined as a group of complex neurodevelopmental disorders, including autism, Asperger syndrome, pervasive developmental disorder not otherwise specified (PDD-NOS), and other relevant symptoms and conditions. A current meta-analysis reported a pooled prevalence of ASD of 0.27% (95% CI: 0.19-35%) in Mainland China, Hong Kong and Taiwan. To take care of children with ASD, parents may suffer from different hardships. A most recent meta-analysis reported that around 31% (95% CI: 24-38%) and 33% (95% CI: 20-48%) of parents of children with ASD were suffer from depression and anxiety, respectively. Another meta-analysis also demonstrated that families of children with ASD experienced more parenting stress than families of children with other disabilities (such as down syndrome, cerebral palsy, or intellectual disability. The psychological distress exact tremendous personal and financial costs on society. Therefore,

it is beneficial for children, parents and the whole society to understand and to find solutions to reduce psychological distress and improve mental well-being in parents of children with ASD.

2. Research objectives

This proposed study aims to develop and implement the mindfulness-based intervention (MBI) using ecological momentary intervention (EMI) and ecological momentary assessment (EMA) platform (EMI/A-MBI) and to assess the effectiveness of this newly developed EMI-MBI on reducing psychological distress in parents of children with ASD. We have three main research questions: (1) what are the daily sources of depression, anxiety and stress in parents of children with ASD and what are the protective factors for resilience? (2) (comparison of the intervention and control group) will the EMI-MBI effectively improve the psychological well-being of parents of children with ASD compared with the control condition? (3) (within the intervention group) will the intensity and frequency of mindfulness practice moderate the participants' cognitive appraisal of daily life events and their psychological distress and mental well-being? In this proposed study, parents' psychological distress will be assessed on the domains of depression, anxiety, stress, and mental well-being indicates participants' life satisfaction and empowerment.

3. Study design

This research adopts a parallel-armed, randomized controlled trial (RCT) design. The intervention group will receive a combination of (1) a time-based system-triggered EMA, which will collect participants' sources and status of depression, anxiety and depression in daily life; (2) EMI delivered MBI, and (3) a follow-up survey of long-term effects of EMI/A-MBI. The participants will first complete a baseline questionnaire, and then participate in EMI via a smartphone application (App) for 8 consecutive weeks and receive the exercise prompts daily. The EMA will include questions of the self-reported feelings of depression, anxiety and depression. After the 8-week EMI-MBI, the participants will be invited to complete a post-experimental survey with similar questions in the baseline questionnaire. Two months after completing the EMI, participants will be contacted to complete a telephone follow-up survey with similar questions in the baseline questionnaire.

4. Intervention and control group

The EMI/A-MBI app will include five main parts, including virtual counselor (a chatbot with pre-set algorism for responding to participants), intervention library, weekly mindfulness practice, assessment bank and daily emotion log. In the eight weeks, the virtual counselor in the chatbot will initiate the conversation every day for three times (morning, afternoon and evening) to check the status of the participants. If the participants responded, the virtual counselor would invite the participants to rate their level of depression, stress and anxiety and then recommend appropriate mindfulness practice in the intervention library, such as 3-minute breathing space or mindful eating. The conversation between the virtual counselor and participants will be in the format of menu list and participants can easily choose from different options. After the participants finished the mindfulness practice, the virtual counselor will check again the participants mental health status. If the participants did not respond to the virtual counselor in the whole day, the EMI/A-MBI app will prompt short survey for psychological distress from assessment bank and recommend mindfulness practice at the end of the day. Participants can also initiate conversation with the virtual counselor at any time of the day and the process will be the same. The participants can also go to the intervention library to find the mindfulness practice that they prefer at that moment. The intervention library will include audios and videos of different mindfulness exercise, such as mindful walking, mindful yoga, or loving and kindness mindfulness. Scripts of the audios and videos will also be provided. The daily emotion log will be prompt to the participants every morning since the second day of the intervention as a visual aid for participants to be aware of the change of their psychological distress and mental well-being.

On every Saturday, the app will prompt reminder for the participants to join the formal 2.5-hour mindfulness practice.
The control group will receive the longitudinal survey exactly the same as the intervention group and 8-week mindfulness-based short-messages sent by the research team on a daily basis. The messages will contain instructions of mindfulness-based practice which will be the same as the intervention group.

3. Human participant(s) involved in the research

[Approximate number, age group, how obtained, and whether the researcher is in a position of power vis-à-vis the participants e.g. teacher-student, employer-employee.]

1. Settings and participants
This study will be conducted in Hong Kong and recruit parents of children with ASD from local organizations for ASD, such as Autism Hong Kong, Autism Partnership Foundation, or Autismilee. The study sites will be the current living environment of the participants for the participants' convenience. The inclusion criteria will comprise (1) parents of children with ASD (children aged between 6-18 and diagnosed with different functional levels of ASD by certified psychologists); (2) own a mobile smartphone with internet access; (3) will stay in Hong Kong during the 8-week EMI study period, and (4) able to read and write in Chinese. The exclusion criteria will include (1) parents diagnosed with depression, anxiety, and stress disorder by certified doctors; and (2) parents who do not live together with their children with ASD on the daily basis.

2. Sample size calculation
The sample size calculation for this study is based on the between-subject effect of the multilevel modelling and multilevel structural equation modelling based on the data from the intervention group. The R package simsem, MASS and lavaan were used for sample size calculation with Monte Carlo simulation run for 1000 times. Parameter of the multilevel modeling used for simulation was based on our pilot study. Results show that 210 participants will be needed to reach the threshold of 80% power to detect the difference, the comparative fit index and the Tucker Lewis Index larger than 0.90, the standardized root mean square residual less than 0.08 and the root mean square error of approximation less than 0.05. In total, 420 (210*2) participants will be needed. We conservatively estimate that around 20% of participants will drop out of the study. Therefore, 526 (263 in each group) will be recruited.

3. Recruitment procedures
Mass emails will be sent to organizations about children with ASD to attract parents interested in the study. Also, online recruitment advertisements will be posted in different online discussion boards. Voluntary participants can directly contact our recruitment staff via telephone, email, or submitting the online application form for a subsequent briefing meeting. Our pilot study has shown that these methods are feasible to recruit a large number of interested participants. After the briefing session, participants' consents will be obtained. The PI is not in a position of power vis-à-vis the participants. The research assistants of this study will not in a position of power or employment vis-à-vis the participants. Only the project team will handle the data collection process.

4. Do your procedures expose your participants to any risk of:

	Yes	No
(a) danger or physical harm	☐	☑
(b) pain	☐	☑

(c)	stress	☐	☑
(d)	fatigue or other form of physical discomfort	☐	☑
(e)	noxious stimulation	☐	☑
(f)	emotional distress or other form of psychological discomfort	☐	☑
(g)	invasion of privacy	☑	☐
(h)	deception	☐	☑
(i)	criminal or civil liability	☐	☑

5. If you have checked any of the "Yes" boxes in 4. above, please –

(a) Estimate the degree of risk involved.

The participants' name will be collected for the baseline, post-test and follow-up assessments. All personal data will be kept strictly confidential and the data will be anonymized for data analysis

(b) Describe the steps you will take to minimize the risk and to protect your participants from it.

Project staff will explain the proposes of collecting participants' names and demographic information. All of the privacy related data collected will be handled by project staff, and will be destroyed with 5 years after completion of the project. Participant has the right to skip or refuse answer demographic information related questions.

(c) How will you explain the risk to your participants?

Please refer to point 5a above

(d) How will you obtain their consent to take part in the research?

Project staff will obtain written consent (Appendix 6 consent form) before distributing questionnaire to the participants

(e) Will there be any payment to the participants?

All participants will be given a HK$50 gift voucher upon completing the baseline survey, post-study survey and 2-month follow-up survey. In addition, participants in the intervention group who have successfully completed all EMI/As in the 8- week will be given an additional HK$50 gift voucher.

(f) Describe how the participants will be debriefed after the study.

The final report will be open access to public.

6. Will you collect names, addresses, or any other details which will make it possible to identify your participants?

☑ Yes ☐ No

If "Yes", please –

(a) Describe the identifiable data you will collect.

Full name of the programme participants will be collected

(b) How will you use these data?

The participants' name will be collected for the baseline, post-test and follow-up assessments. All personal data will be kept strictly confidential and the data will be anonymized for data analysis

(c) How will you dispose of these data?

All data will be kept strictly confidential, and they will be destroyed 5 years after the completion of the project

(d) What procedures will you follow to make sure that your participants cannot be identified?

The project will assign a code to all the participants, and the dataset will be deidentified. The code name match list will be separated from the datasets and kept confidential with limited access of PI and delegated persons.

7. Declaration by Principal Investigator

a) I undertake to exercise reasonable care to ensure that the proposed research is conducted in a manner that is consistent with international standards of ethical practice.

b) The informed consent procedures will be followed.

c) I undertake not to proceed with data collection/analysis before I receive the approval for this application.

ORKT Enquiry: Tel 2616 7683 Email: orkt@LN.edu.hk
