# Supplementary material for: The mechanism and effectiveness of mindfulness-based intervention for reducing the psychological distress of parents of children with autism spectrum disorder: A protocol of randomized control trial of ecological momentary intervention and assessment
Source: PLoS One. 2023 Sep 13;18(9):e0291168. doi: 10.1371/journal.pone.0291168 (PMC10499232; doi:10.1371/journal.pone.0291168)
Supplement: S4 File — (PDF) [file pone.0291168.s005.pdf]

S5 File. Informed Consent Form for Adult

The mechanism and effectiveness of mindfulness-based intervention for reducing the psychological distress of parents of children with autism spectrum disorder: A randomized control trial of ecological momentary intervention and assessment

You are invited to participate in a research study conducted by Dr. WANG Qi in the School of Graduate Studies at Lingnan University. This proposed study aims to develop and implement the mindfulness-based intervention (MBI) using ecological momentary intervention and ecological momentary assessment platform (EMI/A-MBI) and to assess the effectiveness of this newly developed intervention on reducing psychological distress in parents of children with autism spectrum disorder (ASD).

Participants will be randomly assigned into the intervention or control group. Participants in the intervention group will be invited to install the ecological momentary intervention and assessment app on their smart phone and participated in the 8-week intervention with mindfulness-based practice. Participants in the control group will receive short-messages about mindfulness-based practice for 8-weeks.

Strict confidentiality will be kept and that the information obtained in the study will be used for research purposes only. The data containing personal identifiers will be kept for 3 years after publication of first paper, and personal identifiers will be removed for long term retention of the research data. Taking part in this study is voluntary. You may choose not to take part or may leave the study at any time without giving a reason. Deciding not to take part or deciding to leave the study later will not result in any penalty or any loss of benefits to which you are entitled.

If you have any questions about the research, please feel free to contact **Dr. Wang (email: vickywang@ln.edu.hk, telephone: 26168617**. If you have questions about your rights as a research participant, contact Office of Research and Knowledge Transfer of Lingnan University.

SIGNATURE

**I ________________________________ (Name of Participant) understand the procedures described above and agree to participate in this study.

**I do not agree to participate in this study.

____

Signature of Participant

Date of Preparation:
Ethics Approval Expiration date:
Ethics Reference Number:
