# Supplementary material for: The mechanism and effectiveness of mindfulness-based intervention for reducing the psychological distress of parents of children with autism spectrum disorder: A protocol of randomized control trial of ecological momentary intervention and assessment
Source: PLoS One. 2023 Sep 13;18(9):e0291168. doi: 10.1371/journal.pone.0291168 (PMC10499232; doi:10.1371/journal.pone.0291168)
Supplement: S1 Table — (PDF) [file pone.0291168.s006.pdf]

S3 Table. Example mindfulness practices that will be embedded in the EMI/A app

**Mindfulness practice**	**Contents**
Mindful breath	Step out of automatic pilot and practice mindful breathing to help oneself leave stressful moments
Mindful eating	Raisin meditation and practice for mindful eating. Mindful eating can help individuals develop greater awareness and appreciation for the experience of eating and promote a more balanced and healthy relationship with food.
Mindful movement	Mindful walking and movement, mindful exercise, plus body scan. Mindful movement can help participants cultivate healthy lifestyles by exercising in busy daily schedules.
Mindfulness of emotions (3-minute breathing space)	Experience emotions with a non-judgmental and accepting attitude. Encourage participants to be aware of their emotions and bodily sensations and observe them without trying to suppress or avoid them. Mindful emotions also involve being able to respond to emotions skilfully rather than reacting impulsively or getting carried away by them.
Mindfulness of thoughts	Individuals learn to observe and become aware of their thoughts in a non-judgmental way. The aim is to develop a more objective perspective on one's thoughts and cultivate greater control over them.
Guided imaginary meditation	Guided imaginary meditation involves using visualization and imagination to promote participants' relaxation, reduce stress, and improve well-being.
Loving-kindness meditation	Loving-kindness meditation aims to cultivate feelings of love, kindness, and compassion towards oneself and others. This practice involves repeating a series of phrases or intentions, such as "May I be happy," "May you be safe," "May all beings be free from suffering," and so on. Loving-kindness meditation can help individuals develop greater compassion, empathy, and connection with others and reduce feelings of anger, resentment, and judgment.
Insight meditation	During insight meditation, individuals typically focus on developing a deep awareness of the present moment and observing thoughts and sensations without judgment or attachment. The practice involves focusing on the breath and bodily sensations and observing the nature of the mind and its contents. During insight meditation, individuals typically focus on developing a deep awareness of the present moment and observing thoughts and sensations without judgment or attachment. The practice involves focusing on the breath and bodily sensations and observing the nature of the mind and its contents.
