# Supplementary material for: The mechanism and effectiveness of mindfulness-based intervention for reducing the psychological distress of parents of children with autism spectrum disorder: A protocol of randomized control trial of ecological momentary intervention and assessment
Source: PLoS One. 2023 Sep 13;18(9):e0291168. doi: 10.1371/journal.pone.0291168 (PMC10499232; doi:10.1371/journal.pone.0291168)
Supplement: Attachment — Submitted filename: Rebuttal letter.docx [file pone.0291168.s007.docx]

**Response to editor**

When submitting your revision, we need you to address these additional requirements.

1. Please ensure that your manuscript meets PLOS ONE's style requirements, including those for **file naming**. The PLOS ONE style templates can be found at

<https://journals.plos.org/plosone/s/file?id=wjVg/PLOSOne_formatting_sample_main_body.pdf> and

<https://journals.plos.org/plosone/s/file?id=ba62/PLOSOne_formatting_sample_title_authors_affiliations.pdf>

*Response:* *Thank you for your reminder. We have formatted the files following PLOS ONE’s style requirement.*

2. We note that you have stated that you will **provide repository information for your data** at acceptance. Should your manuscript be accepted for publication, we will hold it until you provide the relevant accession numbers or DOIs necessary to access your data. If you wish to make changes to your Data Availability statement, please describe these changes in your cover letter and we will update your Data Availability statement to reflect the information you provide

*Response: Thank you for bringing this to our attention. Since this is a study protocol and data collection is estimated to take 1-1.5 years, we are unable to provide repository information for the data at this time. However, we plan to release all of our data once the study is complete and manuscript writing has finished.*

3. Your **ethics statement should only appear in the Methods section of your manuscript**. If your ethics statement is written in any section besides the Methods, please delete it from any other section.

*Response: Thank you so much for your reminder. We have moved the ethics statement at the end of the methods section of the manuscript, above the section on data management. We also deleted the sentence about the ethics statement in the data management section.*

4. Please include **captions for your Supporting Information files** at the end of your manuscript, and update any in-text citations to match accordingly. Please see our Supporting Information guidelines for more information: <http://journals.plos.org/plosone/s/supporting-information>.

*Response: Thank you so much for this reminder. We have included a list of the captions of the supporting information files at the end of the manuscript, before the reference section. We also updated all in-text citations to match accordingly.*

**Comments to the Author**

1. Does the manuscript provide a valid rationale for the proposed study, with clearly identified and justified research questions?

The research question outlined is expected to address a valid academic problem or topic and contribute to the base of knowledge in the field.

Reviewer #1: Partly

Reviewer #2: Yes

*Response: Thank Reviewer #1’s for the comments. We have made revisions in the method and analysis part to address Reviewer #1’s detailed concerns with the research design. Point-point response is listed below.*

2. Is the protocol technically sound and planned in a manner that will lead to a meaningful outcome and allow testing the stated hypotheses?

The manuscript should describe the methods in sufficient detail to prevent undisclosed flexibility in the experimental procedure or analysis pipeline, including sufficient outcome-neutral conditions (e.g. necessary controls, absence of floor or ceiling effects) to test the proposed hypotheses and a statistical power analysis where applicable. As there may be aspects of the methodology and analysis which can only be refined once the work is undertaken, authors should outline potential assumptions and explicitly describe what aspects of the proposed analyses, if any, are exploratory.

Reviewer #1: Yes

Reviewer #2: Yes

3. Is the methodology feasible and described in sufficient detail to allow the work to be replicable?

Descriptions of methods and materials in the protocol should be reported in sufficient detail for another researcher to reproduce all experiments and analyses. The protocol should describe the appropriate controls, sample size calculations, and replication needed to ensure that the data are robust and reproducible.

Reviewer #1: Yes

Reviewer #2: Yes

**4. Have the authors described where all data underlying the findings will be made available when the study is complete?**

The [PLOS Data policy](https://journals.plos.org/plosone/s/materials-and-software-sharing) requires authors to make all data underlying the findings described in their manuscript fully available without restriction, with rare exception, at the time of publication. The data should be provided as part of the manuscript or its supporting information, or deposited to a public repository. For example, in addition to summary statistics, the data points behind means, medians and variance measures should be available. If there are restrictions on publicly sharing data—e.g. participant privacy or use of data from a third party—those must be specified.

Reviewer #1: No

Reviewer #2: Yes

*Response: Thanks for Reviewer #1’s comments. We have restated our data availability at the data management section: Before the conclusion of the research project, no data will be shared, and data will be available upon request to the corresponding author after the completion of the study. Once the study is complete and manuscript writing has finished, we will also upload all of our anonymous data to a data repository website.*

5. Is the manuscript presented in an intelligible fashion and written in standard English?

PLOS ONE does not copyedit accepted manuscripts, so the language in submitted articles must be clear, correct, and unambiguous. Any typographical or grammatical errors should be corrected at revision, so please note any specific errors here.

Reviewer #1: Yes

Reviewer #2: Yes

**6. Review Comments to the Author**

Reviewer #1:

1. In the "Outcomes" section (line 338), it is necessary to provide a clear distinction between primary and secondary outcomes. While the abstract only mentions depression scale as the primary outcome, it is essential to address the inclusion of anxiety and stress as secondary outcomes. The hypotheses and subsequent analyses should align with the designation of primary and secondary outcomes to ensure consistency throughout the study.
   *Response: In the abstract, we stated that we will utilize the depression anxiety stress scale to assess our primary result. Since this single scale can measure all three factors of depression, anxiety, and stress simultaneously, we have decided to incorporate them as our primary outcome measures. We have added the description of the primary and secondary outcomes in the outcomes section:*

*“The primary outcome will be participants’ psychological distress measured by the depression anxiety stress scale. The secondary outcomes will include participants’ subjective well-being, measured by the satisfaction with life scale, and level of resilience, measured by the psychological empowerment scale.”*

2. The primary aim of the study is to examine whether the intervention reduces psychological distress, as stated in the first hypothesis. Consequently, the primary analyses should focus on comparing the two treatment groups using appropriate statistical tests such as t-tests or linear mixed models. The use of Structural Equation Modeling (SEM) for the first research question is unclear and may not align with the primary outcome of reducing psychological distress.
   *Response: Thank you so much for pointing out this important issue. We have revised the data analysis section as follows:*

*“To answer the first and second questions, first of all, the effect size (Heges’ g) of the outcome for both the intervention and control groups will be calculated. Subgroup analysis will be conducted when necessary. Next, linear mixed models will be conducted to compare the effectiveness of the mindfulness intervention between the two treatment groups. Linear mixed models are particularly suitable for analyzing data collected via ecological momentary assessment, as they can account for the nested and repeated nature of the data. The main predictor variable will be treatment group (intervention vs. control), and the model will also include baseline DASS scores and any relevant demographic variables (e.g., age, gender) as covariates. The linear mixed model will include random intercepts and slopes for each participant to account for individual differences in the baseline scores and the rate of change in scores over time. The treatment group effect will be assessed using a fixed effect, and the model will also include an interaction term between treatment group and time to investigate any differential effects of the intervention over time.”*

3. The sample size calculation should be based on the comparisons between the two treatment groups, rather than derived from SEM considerations. It is crucial to ensure that the sample size is adequately powered to detect differences in the primary outcomes between the treatment groups.

*Response: Thank you so much for your guidance. We have revised the method to calculate sample size and revised it as follows:*

*“The sample size calculation for this study is based on the linear mixed model that will be adopted to answer research questions 1 and 2 based on the repeated ecological momentary assessment data from the intervention and control group and the effect size of 0.29 from previous studies (unpublished manuscript). The R package sjstas (45), and pwr (46) were used for sample size calculation. The results indicate that a total of 536 participants will be required to detect a significant difference at an alpha level of 0.05 and a power of 80%. Accounting for potential dropouts, we conservatively estimate that 20% of participants may not complete the study. Therefore, we plan to recruit 670 participants (335 in each group).”*

4. Line 460-462: The analysis procedure described for the second research question is not clearly presented. It is important to note that the effect size is a measure of the difference between two groups and cannot be directly "compared" between the groups.
   *Response: Thank you so much for pointing out this important issue. We have revised the data analysis section as follows:*

*“To answer the first and second questions, first of all, the effect size (Heges’ g) of the outcome for both the intervention and control groups will be calculated. Subgroup analysis will be conducted when necessary.”*

5. How will missing data that is not at random (MNAR) be dealt with?

*Response: Thank you so much for pointing out this important issue. We have added in the main text that:*

*“The characteristics of the observed data and the missing data will be compared to determine whether there are any systematic differences. If tested that the missing data will be missing not at random, imputation methods, such as multiple imputation (MI) or maximum likelihood estimation (MLE), will be adopted to estimate the missing values based on the observed data and the relationships between variables. Moreover, sensitivity analysis will be adopted to assess the impact of the missing data on the study results and to determine the robustness of the findings.”*

Reviewer #2:

1. The “Introduction” part is lengthy. Most part of it consists of theoretical discussion. There should be a separate “Literature Review” Part. The paper should demonstrate an adequate understanding of the relevant literature in the field and cite an appropriate range of literature sources.

*Response: Thank you so much for your suggestion. We have cut some sentences in the introduction part to make it more concise. To follow the manuscript organization requirement of the journal (https://journals.plos.org/plosone/s/submission-guidelines), we kept the current structure of abstract-introduction-method and results-discussion. Thank you for your understanding.*

2. The “Methods” part is well-written. The methods are employed appropriately and able to meet the objectives of the paper. The “Results” and “Discussion” parts are well-written.

*Response: Thank you. We appreciate the compliment.*

3. The study should address “policy implications” and “conclusion”.

*Response: Thank you so much for your suggestion. We have added the policy implications and conclusion sections at the end of the manuscript on page 23-24.*

*“Despite the limitations, the findings of this study may highlight the significance of digital health interventions, such as EMI/A, in the field of mental health care. It may provide evidence for policymakers to support initiatives that educate and train healthcare providers on the use of such tools. This may include funding for research, subsidies for application development, or assistance with integrating these technologies into the existing healthcare infrastructure. In addition, if this study demonstrates the efficacy of MBI, it may be necessary to implement policies to ensure that these interventions are widely accessible. This could involve the incorporation of MBI in health insurance coverage or the development of public programs that serve to disseminate mindfulness techniques to parents of children with ASD and possibly other populations experiencing psychological distress.”*

*“In conclusion, this study aims to shed light on the effectiveness of MBI delivered through digital means, specifically the EMI/A app, in alleviating psychological distress among parents of children with ASD. If the findings are positive, they could signal a turning point in how MBI is delivered, moving away from traditional in-person methods towards more flexible and personalized digital interventions. Ultimately, the potential benefits of this study could extend beyond the participant group to potentially aid individuals struggling with other psychological issues, marking a significant step forward in mental health care.”*
